# Bullous pemphigoid in a previously healthy adolescent: a case report and literature review

**DOI:** 10.1097/MS9.0000000000000995

**Published:** 2023-07-22

**Authors:** Narmeen Giacaman, Rawan Sami N. Abusaada, Salem M. Tos, Mohammad G. Ibdah, Adam M. Reid Mahagney, Asmaa Rjoob, Musallam Abukhalil, Hamza Salim, Basel Musmar, Sufyan Zuwahreh

**Affiliations:** a College of Medicine, Al-Quds University, Abu Dis; b Internal Medicine Department, Beit-Jala Governmental Hospital, Bethlehem; c Faculty of Medicine, Islamic University of Gaza, Gaza; d An-Najah National University, Nablus, Palestine; e The Hebrew University, Hadassah Medical School, Jerusalem, Israel

**Keywords:** bullae, bullous pemphigoid, case report, clinical dermatology

## Abstract

**Introduction:**

Bullous pemphigoid (BP) is considered the most common bullous autoimmune disorder, characterized by autoantibodies directed against hemidesmosomes in the skin and mucous membranes. It usually affects elderly individuals in the sixth through eighth decades of life, with an average age at onset of 65 years. Only a few cases have been reported in children and teenagers.

**Case presentation:**

Herein, we report a 17-year-old boy who presented with a pruritic vesicular rash on his arms and legs accompanied by erythema. He was treated at the beginning with topical lotion and acyclovir, but the rash kept deteriorating and eventually bullae appeared, involving also his mouth. A dermatologist was consulted and diagnosed him with BP, and he was treated accordingly.

**Discussion:**

BP is the most prevalent autoimmune bullous illness, caused by autoantibodies against hemidesmosomes in the basement membrane of skin and mucosal surfaces, which in turn attract immune cells, including T-cells and neutrophils, and activate them, which causes damage to and separation of keratinocytes, resulting in the bullous formation. Diagnosis can be accomplished by recognizing clinical symptoms supported by histopathological and immunofluorescence testing. Steroids, whether topical or systemic, are the cornerstone treatment; depending on the extent of the disease, other immunosuppressant drugs can be used as a second line.

**Conclusion:**

BP manifestations are polymorphic; physicians should keep in mind that they may present with non-bullous, pruritic lesions, which may persist for some days to several months before bullae appear. Although this disease is rare in the young population, it should be considered in the differential diagnosis of bullous lesions.

## Introduction

Highlights

- Bullous pemphigoid is rarely seen in adolescence.
- The cutaneous manifestations of bullous pemphigoid are polymorphic.
- It can be misdiagnosed on initial presentation before bullae appear.

In 1953, Lever introduced the term pemphigoid to describe a disease characterized by bullous formation due to subepidermal detachment to distinguish it from pemphigus, an intraepidermal blistering disorder brought on by acantholysis.

Only few cases of bullous pemphigoid (BP) were reported in children and teenagers in the literature, as this disease mainly affects the elderly in their eighth decade of life and has no preference for either gender^1^. In this case, we describe a patient with free past medical and surgical history that first presented with pruritic vesicular rash surrounded by erythema on his arms, legs, and trunk for a few days, followed by diffuse skin involvement of bullous lesions. He was treated mainly with topical and systemic corticosteroids, which led to significant improvement. On follow-up, his lesions healed, and he was back to his normal life.

## Case presentation

**Figure 1:**
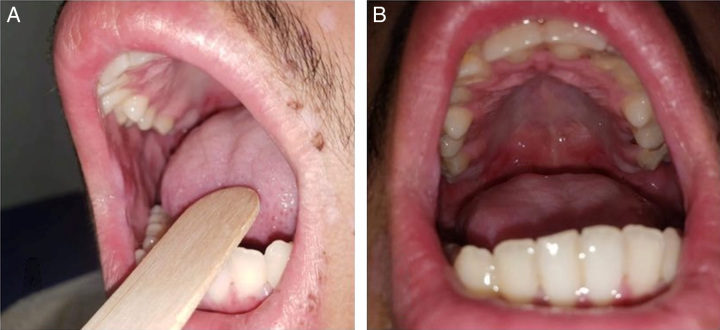
Painful mouth lesions involving the oral mucosa and the soft palate.

A 17-year-old boy complained of pruritic vesicular rash surrounded by erythema on his arms, legs, and trunk for few days. He was seen by his family doctor and was diagnosed with chickenpox caused by varicella-zoster virus. The doctor gave him symptomatic treatment (calamine lotion) for the pruritis and acyclovir. Two days later, the rash kept getting worse and bullae started to appear; he also developed painful mouth lesions (Fig. 1). The patient was seen by a dermatologist in his clinic who suspected bullous pemphigoid and sent him to be admitted to our hospital for treatment as the patient could not have anything by mouth.

**Figure 2:**
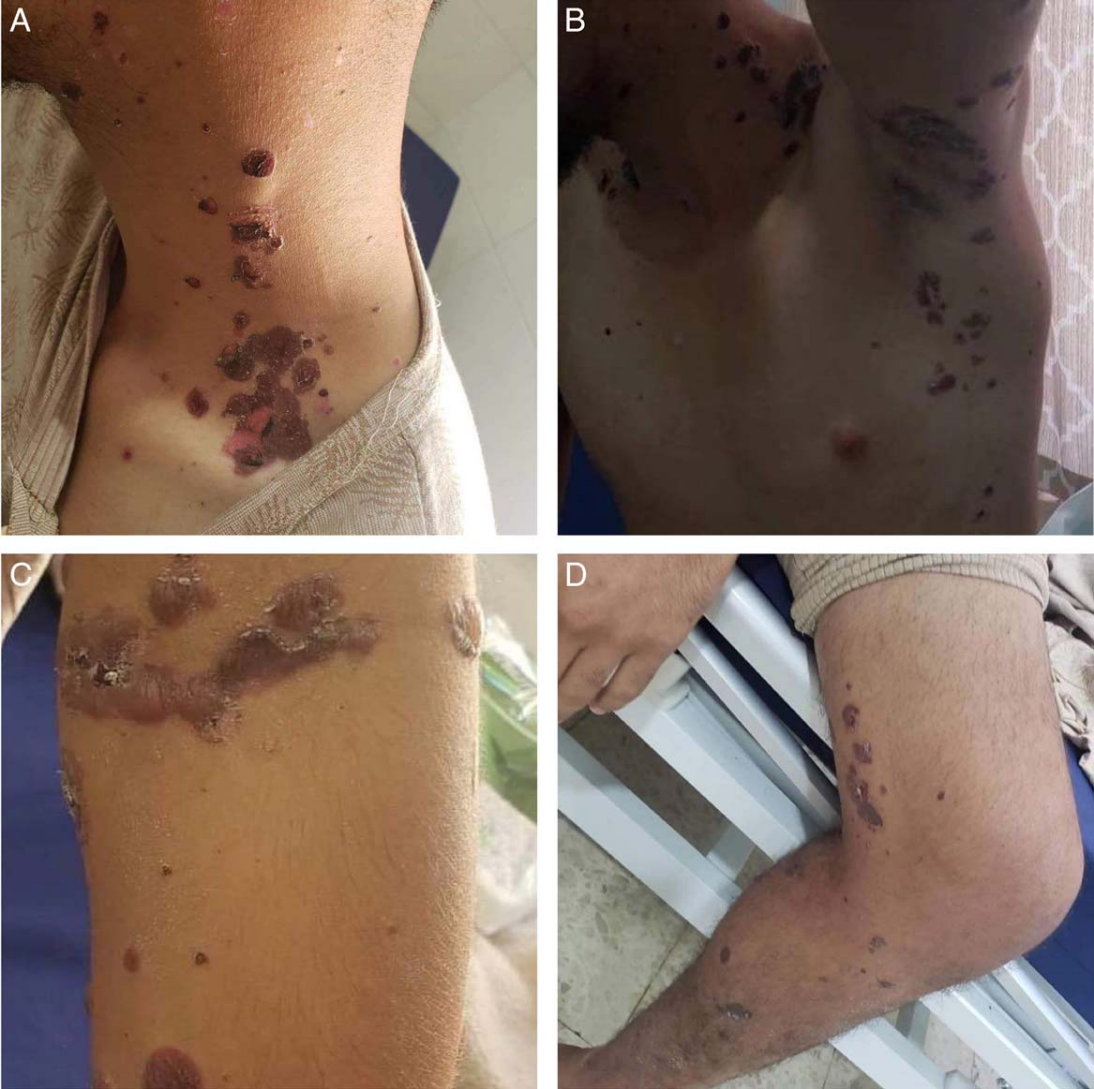
Diffuse skin involvement by bullous pemphigoid. Pictures taken after 4 days in treatment.

**Figure 3:**
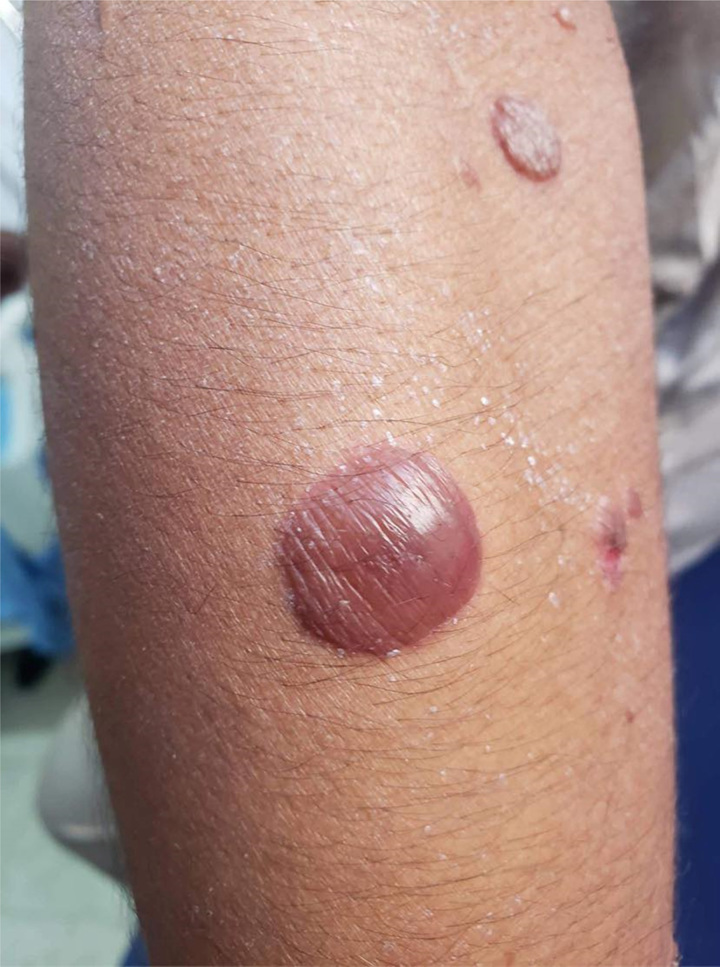
Zoomed in the picture of a bullous.

On admission, the patient had stable vital signs but looked dehydrated with cracked lips and dry skin. Diffuse skin involvement was seen (Figs 2, 3) including axillae, arms, legs, trunk, oral mucosal membrane, and the groin area. Nikolsky sign was negative on examination. Lab tests were all in the normal range for his age, including complete blood count, Thyroid function tests, basic metabolic panel, liver function tests, and kidney function tests. So, he was started on intravenous (i.v.) fluids to prevent dehydration, pantoprazole 40 mg i.v., amoxicillin/clavulanic acid to prevent secondary skin infection, triamcinolone cream for his oral lesions, clobetasol lotion (corticosteroid) for his skin lesions for 2 weeks and methylprednisolone 80 mg IV*1 to be converted to prednisolone 40 mg once oral intake is tolerated.

Two punch biopsies were taken and the diagnosis of BP was confirmed by direct immunofluorescence and histopathological examination.

Few days into treatment, the patient’s lesions started healing and he could tolerate soft food intake. He was discharged on prednisolone 40 mg per day to be tapered for the next 2 months by 5 mg per week before stopping the drug. A week later, the patient had improved significantly, and his lesions were healing well with no new lesions. On follow-up, his lesions healed and he was back to his normal life.

The patient presented in this case had free medical and surgical history, no family history of autoimmune diseases, and has not taken any drug for the past few months since the lesions started.

This work has been reported in line with the Surgical CAse REport (SCARE) criteria, which is used by authors, journal editors, and reviewers to increase the robustness and transparency in reporting medical and surgical cases^2^.

## Discussion

The most prevalent autoimmune bullous illness is BP, which is characterized by autoantibodies against hemidesmosomal proteins of the skin and mucous membranes^3^. A distinguishing aspect of the illness is the presence of tissue-bound and circulating autoantibodies directed against structural components of the hemidesmosomes that connect basal keratinocytes to the basement membrane (BM)^1^.

Neutrophil chemotaxis and BM zone destruction are caused by a dysregulated T cell immune response and the production of immunoglobulin (Ig)G and IgE autoantibodies against hemidesmosomal proteins (BP180 and BP230)^4^.

BP is a disease that mainly affects the elderly and is quite rare in children and adolescents. Dr Patsatsi *et al*. have published an impressive review about BP in adolescence. The review included nine cases of BP in adolescents, describing aspects as skin lesions seen, mucosal involvement, treatment, disease course, and others in a very comprehensive and tidy table^5^. In addition to the cases, we decided to report our case as a case of BP diagnosed in an adolescent to emphasize the importance of keeping BP in any adolescent presenting with vesicles or bullae. And to remind clinicians that initially, bullae may not be present and patients could be misdiagnosed, as our patient was first misdiagnosed and given treatment for chickenpox.

The diagnosis of BP is based on a combination of clinical characteristics, histological findings, and immunofluorescence results; histopathological examination reveals eosinophilic spongiosis or a subepidermal detachment with eosinophils^6^, immunofluorescence as direct or indirect tests identify linear IgG and/or C3 deposition at the BM zone^7^, and ELISA (enzyme-linked immunosorbent assay) measure circulating autoantibodies against BP180 and/or BP230. Direct immunofluorescence test is considered the gold standard for diagnosis^8^.

In the case presented to you, two punch biopsies were taken to confirm the diagnosis. On histopathological examination, subepidermal acantholytic reaction with blister formation was seen, mostly in keeping with BP. Moreover, using the direct immunofluorescence test, linear deposition of IgG and complement C3 was seen along the dermal–epidermal junction.

Cutaneous symptoms of BP are variable. The condition frequently starts with a non-bullous, pruritic phase that can last from a few days to many months and is occasionally the first symptom of BP^9^. During this stage, urticarial or excoriated lesions, eczematous plaques, or prurigo-like lesions occur, making accurate diagnosis difficult^10^. As was seen with the patient in this case, the first presentation consisted of a pruritic vesicular rash surrounded by erythema without bullae that was misdiagnosed as chickenpox before bullae started to appear.

Classical BP is clinically distinguished by 1–3 cm diameter tense, serous, or hemorrhagic bullae on erythematous or otherwise normal skin^11^. Severe pruritus is observed in virtually all patients. The condition has a symmetric distribution, and the lower abdomen, flexor surfaces of the limbs, groins, and axillae are common predilection areas^1^.

The goal of BP treatment is to halt the progression of new lesions, promote cutaneous healing, and alleviate pruritus. Because BP mostly affects the elderly, therapy must be adjusted to the patient’s comorbidities and capacity to self-care in order to minimize probable consequences and increased morbidity and mortality^12^.

The German guideline recommends stage-adjusted treatment depending on the affected skin surface area as the best practical method for treating BP. If involvement is less than 10%, only topical glucocorticoid monotherapy is recommended; if participation is greater than 30%, a combination of topical and systemic glucocorticoids is recommended. Between 10 and 30% of the time, systemic therapy is an option. Systemic glucocorticoids should be used in severe hypertension and tested in mild hypertension^13^. In the case presented, a combination of topical and systemic glucocorticoids was used as more than 30% of the skin surface area was affected.

The recommendations prescribe or may explore the following additional medications: cyclosporine, cyclophosphamide, plasmapheresis/immunoapheresis, sulfonamides, topical tacrolimus, TNF (tumor necrosis factor) inhibitors, and other biologic agents^12-14^. According to the updated S2K guidelines for BP management, Immunosuppressive treatments, such as methotrexate, azathioprine, mycophenolate mofetil, or mycophenolate acid, may be advised in the event of contraindications or resistance to corticosteroids. In treatment-resistant cases, intravenous immunoglobulins and B-cell-depleting therapy may be explored. Dupilumab and omalizumab have lately demonstrated encouraging outcomes too^15^.

## Conclusion

In conclusion, although uncommon at a young age, BP must be kept in mind when putting the list of differential diagnoses of a young patient presenting with vesicles or bullae.

In addition, clinicians should keep BP in mind when facing cutaneous lesions, as the cutaneous manifestations of BP are polymorphic. So, it can initially present as non-bullous, pruritic lesions which may persist for some days to several months before bullae appear and sometimes remain the only sign of BP.

## Ethical approval

The study is exempt from ethical approval in our institution.

## Consent

Written informed consent was obtained from the patient’s parents for the publication of this case report and accompanying images. A copy of the written consent is available for review by the Editor-in-Chief of this journal on request.

## Sources of funding

No funding or grant support.

## Author contribution

N.G.: study concept or design; N.G., R.S.N.A., S.M.T., M.G.I., A.M.R.M., A.R., M.A., H.S., and B.M.: writing the manuscript; N.G., S.M.T., and S.Z.: review and editing the manuscript.

## Conflicts of interest disclosure

The authors declare that they have no conflicts of interest.

## Research registration unique identifying number (UIN)

Not applicable.

## Guarantor

Sufyan Zuwahreh.

## Provenance and peer review

Not commissioned, externally peer-reviewed.
